# Supplementary material for: Detecting patterns of species diversification in the presence of both rate shifts and mass extinctions
Source: BMC Evol Biol. 2015 Aug 11;15:157. doi: 10.1186/s12862-015-0432-z (PMC4530483; doi:10.1186/s12862-015-0432-z)
Supplement: Additional file 1 — Details of the comparisons between our backward and the forward-like algorithms used to perform the simulations. [file 12862_2015_432_MOESM1_ESM.pdf]

Detecting patterns of species diversification in the presence of both rate shifts and mass extinctions

Sacha Laurent, Marc Robinson-Rechavi and Nicolas Salamin

Department of Ecology and Evolution, University of Lausanne, Biophore, 1015, Lausanne, Switzerland
Swiss Institute of Bioinformatics, Quartier Sorge, 1015 Lausanne, Switzerland

We compared our backward and forward-like algorithms to assess if the backward approach could introduce a potential bias in the branching times distribution. This could then have an effect on the rate shifts that are inferred by Medusa. We therefore did a series of simulations to test the differences between the two algorithms.

The Supplemental Figure 1 show that for every species-numbered tree, forward and backward algorithm yields comparable branching times densities. As both methods tested in our paper take branching times as only input, we conclude that the way we simulated our trees in our paper as little chance to bias the results in anyway.

The Supplemental Figure 2 shows the results for 50 trees of 500 and 200 species. We observe that in both cases, similar numbers of diversification shifts were found by Medusa. Regarding distances between simulated and identified shifts by Medusa, most of the events found were situated at a close distance of the real event, even though we observe a slight tendency for a lower precision in the forward-like algorithm. This could be an effect of the overall size of the tree (as the forward-like would always be smaller than the 200 or 500 species sharp backward tree). Indeed, as we see in the Fig 3 of the manuscript, the effect of the overall tree size is low in general, but there is a slight difference for small clades and 200/500 species trees, that could explain the discrepancy we are seeing here. In general, we feel that these new simulations prove the small effect that our simulation schemes has in distorting the main results of our paper.

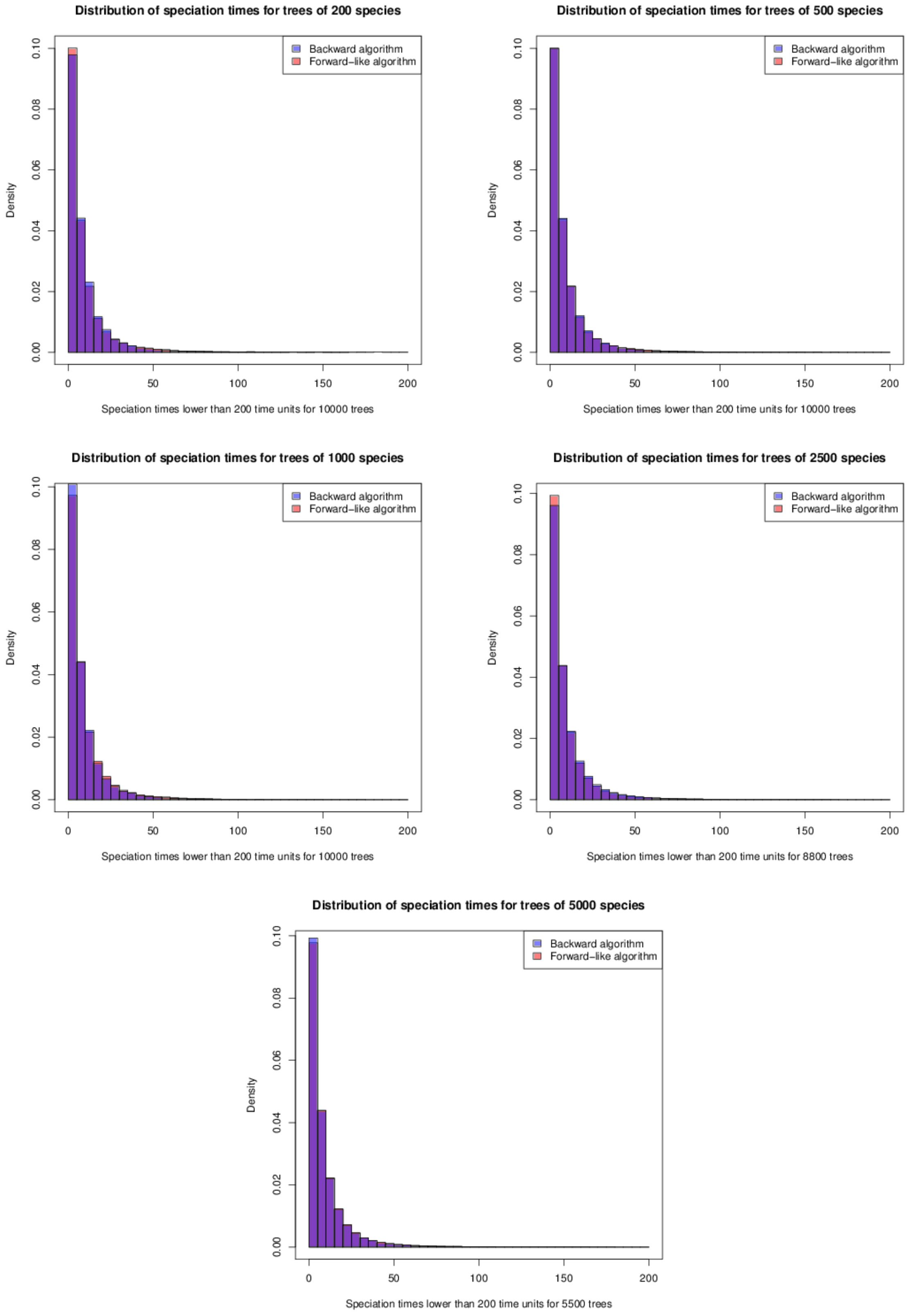

Suppl. Figure 1: Comparison of our backward and forward-like algorithms with trees of different sizes when analysed with Medusa. We simulated 10000 trees of size 200, 500, 1000, 2500 and 5000 tips.

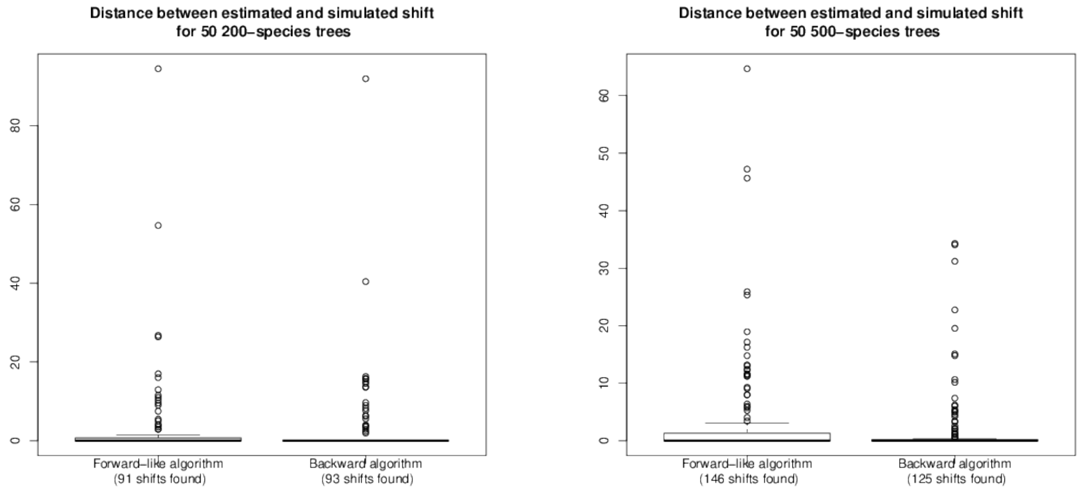

Suppl. Figure 2: Differences in branching times between trees obtained with our backward or the forward-like algorithms. We simulated 50 trees with either 200 (left panel) or 500 (right panel) tips.
